# Primary Cavity Excavators in Community‐Managed Lowland Forest of Nepal: Woodpecker Assemblage, Nest‐Tree Preference, and Its Implications for Forest Management

**DOI:** 10.1002/ece3.74611

**Published:** 2026-11-10

**Authors:** Nikeet Pradhan, Sandeep Regmi, Bikash Adhikari

**Affiliations:** ^1^ Faculty of Forestry Agriculture and Forestry University Hetauda Makwanpur Nepal; ^2^ Department of Biological Sciences University of Lethbridge Lethbridge Alberta Canada; ^3^ The Himalayan Conservancy Kirtipur Nepal; ^4^ Southeast Asia Biodiversity Research Institute, Chinese Academy of Sciences and Center for Integrative Conservation, Xishuangbanna Tropical Botanical Garden Chinese Academy of Sciences Mengla Yunnan China; ^5^ University of Chinese Academy of Sciences Beijing China

**Keywords:** cavity nesters, community forest, distance sampling, keystone species, snag

## Abstract

Woodpeckers are keystone species playing a crucial role as ecosystem engineers, with their highest species richness and diversity in the tropical and subtropical regions. Although most research on the species' ecology originates from the temperate regions, it leaves a substantial gap in woodpecker assemblage and nesting ecology in tropical and subtropical areas. Our study fills this gap in the subtropical region of South Asia, exploring woodpecker diversity, abundance, and factors influencing cavity nest occurrence in the lowland sal *Shorea robusta* forests of Nepal. The study was carried out in May and June 2024 in the Pathlaiya Community Forest (CF), with 24 line transects established. Each transect was surveyed twice for woodpecker abundance and nesting cavities, with data gathered on the perpendicular distance of woodpecker observation from the transect, tree characteristics, and nesting variables. Distance sampling analysis was used to estimate an overall woodpecker density and detection probability in the study area, which were found to be 0.54 ± 0.07 birds/ha and 0.79 ± 0.05, respectively. Overall, 51 individuals from nine woodpecker species were recorded. In Nepal's lowland sal forests, the nesting occurrence of woodpeckers was significantly influenced by factors: canopy cover, tree condition and tree Diameter at Breast Height (DBH), with emphasis on sal and jamun *Syzygium cumini* as preferred tree species. It was found that woodpecker cavity nest occurrence depends substantially on mature forest structure for nesting, highlighting the importance of retaining adequate amount of snags and larger trees, aligning with sustainable forest management practices. These findings emphasize community‐managed tropical sal forests as important habitats for primary cavity excavators, particularly Picidae. Our study highlights the importance of retaining large native trees, snags, and cavity‐bearing tree species in forest management plans to support woodpeckers and the larger cavity‐nesting species in South Asia.

## Graphical abstract

Woodpeckers are keystone species playing an important role in the ecosystem as primary cavity excavators. Our study fills a crucial gap in woodpecker assemblages and nesting‐tree preferences in sub‐tropical forests in South Asia, which remain limited.

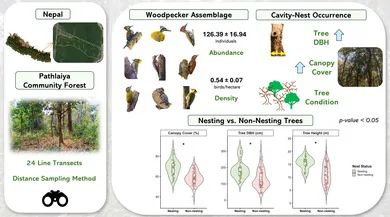

## 1 Introduction

Cavity‐nesting birds represent a functionally distinct guild uniquely dependent on tree cavities as a vital ecological resource to meet their breeding and roosting needs (Bonaparte et al. 2024). Tree cavities are a limited forest resource in most forest ecosystems on which a plethora of non‐excavator species (van der Hoek et al. 2017) rely, which makes them a key resource regulating species interactions, competition, and community structure (Kosinski and Winiecki 2004). In this complex nest‐web dependency, primary excavators are the foundations of this network, creating essential forest habitat resources (Maya‐Elizarrarás et al. 2025). Primary excavators such as woodpeckers (Picidae) are considered keystone species and ecosystem engineers owing to their ability to construct tree cavities, which provide essential resources for other species to roost and nest (Bednarz et al. 2004; Mikusiński 2006). Additionally, their ecological function extends beyond cavity excavation, as their tree‐drilling activity facilitates sap‐consuming organisms and also aids in fungal dispersal (Baral 2023; Catalina‐Allueva and Martín 2021; Robles and Martin 2014). These crucial ecosystem roles of woodpeckers actively shape habitat and resource availability for multiple taxa, which influence the avian community assemblage. Consequently, woodpecker richness is often perceived as an indicator of overall forest bird richness, making them effective umbrella species for conservation planning (Menon and Shahabuddin 2021; Virkkala 2006). However, their reliance on larger and taller trees, particularly snags, for foraging and breeding renders them highly vulnerable to logging and forest degradation (Drever et al. 2008; Menon and Shahabuddin 2021).

Human modification of forest landscapes by the removal of large, mature trees and snags by intensive logging depletes the specialized nesting and foraging substrate for cavity nesters (Ke et al. 2023). This is directly evident from their global population decline and potential extinction risk in many regions (Mikusiński 2006). Removal of suitable tree species from a managed forest stand forces woodpeckers to use the limited trees more intensely, indicating resource scarcity and the emergence of an ecological bottleneck. This restriction increases resource competition for cavities and diminishes breeding opportunities, leading to global population decline and extinction risks in numerous habitats (Mikusiński 2006). Not only woodpeckers, but these activities also threaten other cavity nesters dependent on these resources, mainly due to silvicultural practices that prohibit the preservation of mature trees and snags (Ke et al. 2023). The deforestation trend is especially alarming in tropical and subtropical regions (Ford et al. 2020), which are the key global hotspots for woodpeckers, but are currently facing the highest rate of habitat loss. The tropical and subtropical regions of Asia, Africa, and South and Central America are global biodiversity hotspots that support the highest woodpecker diversity yet are facing a higher rate of deforestation. Despite hosting the majority of Picidae species and increasing threats, these regions remain severely understudied. Most of the published research on woodpeckers is from North America and Europe, which only accounts for 17% of the Picidae species (Mikusiński 2006). In Asia, studies on woodpeckers have primarily focused on abundance and richness (Kumar et al. 2014; Lammertink 2004), conservation status (Kumar et al. 2011), distribution and ecology (Santharam 2003; Styring and Hussin 2004), and checklists. However, a critical gap remains regarding community assembly, nesting tree preference, and response to fine‐scale habitat associates. Understanding species assemblage patterns is critical for determining how sympatric woodpecker species allocate resources and coexist within the same environment (Shafaeipour et al. 2024). On the other hand, woodpecker nesting preference is determined by multiple layers of spatial scales, from the specific nest tree to the local environment (Purcell and McGregor 2021), which is important to assess, as it can vary across these levels.

Nepal provides an ideal natural laboratory to address these gaps, as the country hosts 25 woodpecker species (2.8% of its avifauna; DNPWC and BCN 2022). Yet quantitative data on their nesting ecology, including tree choices, tree attributes, and associated ecological and anthropogenic factors in tropical forests, remain virtually absent. To address these gaps, our study aims to quantify the structure of the woodpecker assemblage and nesting preferences in the lowland forests of southern Nepal. Although woodpeckers are recognized as major cavity excavators, their function in subtropical community‐managed forests in South Asia is not well understood. This gap is crucial since community forests are increasingly managed for both wood production and conservation of biodiversity. Therefore, we aim to: (1) estimate the abundance, density, and species richness of the woodpeckers, (2) determine nesting tree preferences, and (3) identify major factors associated with woodpecker cavity‐nest occurrence. We hypothesize that the nesting tree selection will not be random, with a preference toward larger trees in the study area.

## 2 Methods

### 2.1 Study Area

This study was conducted in the Pathlaiya Community Forest (CF) situated in the lowland of Madhesh Province, Nepal (Figure 1). The CF is located to the east of the Parsa National Park and is adjacent to the national park. The CF is at an elevation of 150 m above sea level and occupies a total area of 232.14 ha. It has a tropical climate with an average temperature of 18.3°C–30.9°C and an annual precipitation of 1967.1 mm (CBS 2019). The CF has a settlement with 412 households in the west, which rely on forest resources for their livelihood. The area's prominent tree species include sal ( *Shorea robusta* ), saj (*Terminalia alata*), jamun ( *Syzygium cumini* ), and barro (*Terminalia bellerica*). The CF's proximity to protected areas makes it rich in prominent wildlife species of lowland Nepal, such as Asian Elephant ( *Elephas maximus* ), Common Leopard ( *Panthera pardus* ), Wild Boar ( *Sus scrofa* ), Barking Deer ( *Muntiacus muntjak* ), Spotted Deer ( *Axis axis* ), and Rhesus Monkey ( *Macaca mulatta* ). Moreover, the dominant avian species in the area include Indian Roller ( *Coracias benghalensis* ), Black‐hooded Oriole ( *Oriolus xanthornus* ), Black‐rumped Flameback ( *Dinopium benghalense* ), and Lineated Barbet ( *Psilopogon lineatus* ).

**FIGURE 1:**
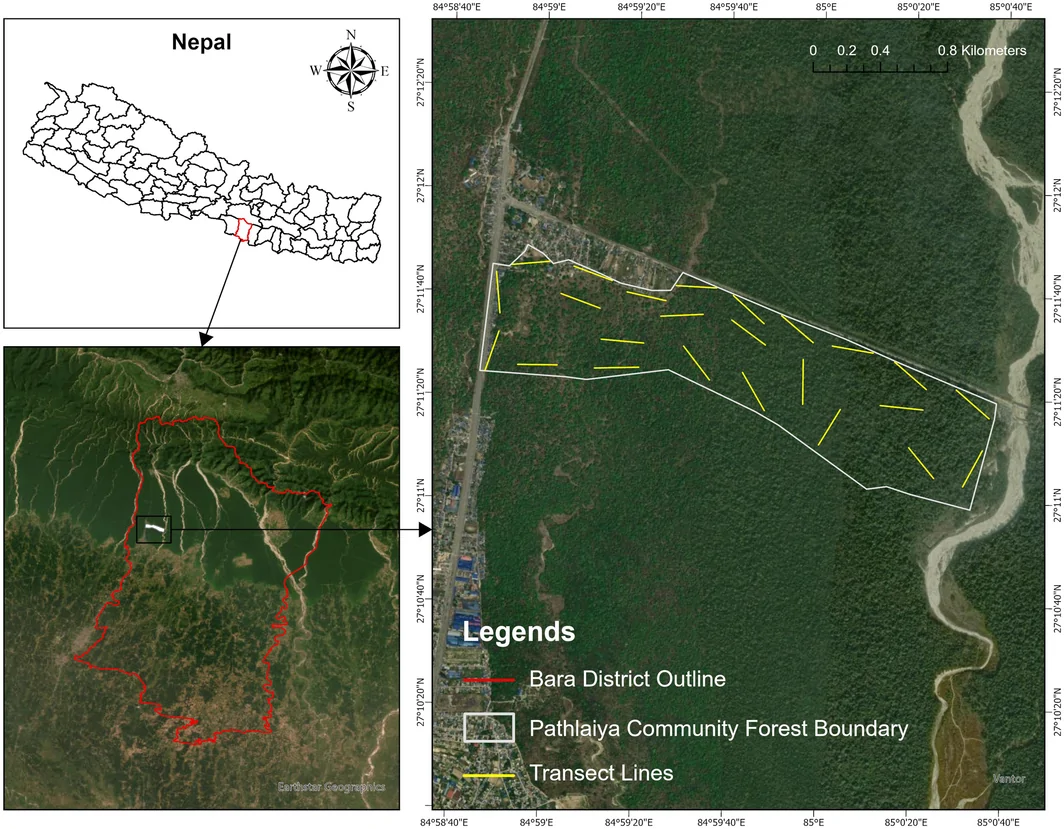
Map of the study area showing the boundary of the Pathlaiya CF with the transect lines where the survey was conducted.

### 2.2 Data Collection

We conducted this study in May–June 2024 (28 days), during the breeding season of most bird species in the Indian Subcontinent (Grimmett et al. 2016). A total of 24 line transects (each 250 m) with a 100 m belt were established in the study area to survey woodpeckers' nesting cavities, conduct distance sampling for woodpecker abundance, and tree sampling. Each transect was separated by at least 100 m and the nearest distance at which each transect had at least one transect was 210 m. Each transect was surveyed twice (once in May and June), overlapping with the most resident woodpecker species' breeding and nesting season in the region. This is a suitable time that increases the observation of active cavities and nesting activity. The data for each survey were treated as an independent event, creating strata to reduce the error that can arise with the multisession distance sampling analysis (Rexstad 2025) if they were pooled together (Buckland 2006). Woodpeckers were surveyed and recorded using a Nikon Acculon binocular, and when a woodpecker was detected in a tree, the perpendicular distance of the woodpecker‐sighted tree from the transect was measured using the measuring tape. When a nesting cavity was identified in a tree, we measured variables like diameter at breast height (DBH), tree height, nest direction, canopy cover, and tree condition. DBH was measured at 130 cm above ground using a measuring tape (Chhetri and Fowler 1996); tree height and nest height were measured using a clinometer; nest direction was measured using a compass; and canopy cover at the tree base was measured using a spherical densiometer. Further, we recorded tree condition (living or snag) as a binomial. Additional variables like distance to water, distance to settlement and distance to forest edge were also calculated from each nesting tree to the closest water source, settlement and forest edge, respectively. Each of these variables was selected as they represent various ecological processes, such as distance to water represents the influence of available water resources, distance to settlement represents the influence of anthropogenic disturbance and distance to forest edge represents the edge‐interior habitat gradient. On both sides of each transect, all tree species (≥ 30 cm DBH were included as trees, DFRS 2015) that did not have any tree cavities were also sampled for DBH, tree height, and canopy cover for a negative control. Different woodpecker species have varying nesting cavity features in terms of entrance size, shape, nest height, and placement on the trunk or branches. However, this was not the sole identification criterion; the woodpecker nesting cavity was observed for adults entering, leaving, drilling, or visiting the cavity to identify the species using the nest cavity. Other primary cavity excavators occurring in the region, particularly barbets such as the Lineated Barbet, were considered during cavity identification. In general, woodpecker nests are more circular than oval, slanted downwards, and have small openings comparable to the woodpecker's body, which was used to distinguish them from other primary cavity nesters (Ćiković et al. 2014).

### 2.3 Data Analysis

Data analysis was done using distance sampling and generalized linear modeling in R software (R Core Team 2026). Distance sampling is commonly used for determining animal density and abundance, which estimates species densities based on appropriate detection rates for all species (Buckland et al. 2001). The detection probability and density of woodpeckers in the Pathlaiya CF were analyzed in R using the Distance package (Miller et al. 2019). All woodpecker species sighted during the survey were pooled and grouped for distance analysis, as sightings of some species were too few for individual analysis. Measured perpendicular distances between the woodpecker‐detected tree (Figure S1) and the transect line were utilized to simulate the detection function. Detection functions were fitted using a variety of potential key functions, including half‐normal and hazard‐rate with null, cosine, and polynomial adjustment variables. Then the model selection was performed based on Akaike's Information Criterion (AIC), with the lowest AIC value regarded as the best‐supported model. The hazard rate with and without any adjustment key function had the lowest AIC value (AIC = 391.3; Table S1; Table S2), so the hazard rate without any adjustment was selected as the best model, as it is the simplest adequate model. Additionally, in the hazard rate without any adjustment, the goodness‐of‐fit test showed no significant difference between the observed and expected detection‐distance distribution (test statistic = 0.0075, *p* = 0.72; Figure S2).

The residuals of the distance model were verified for spatial autocorrelation using Moran's *I* (Lichstein et al. 2002) from the density surface model for potential pseudo‐replication, which may arise from close transect lines. Residuals were analyzed independently for each visit and then averaged across visits to produce a transect‐level measure of residual variance. Spatial proximity was defined as the shortest euclidean distance between entire transect geometries using the sf package (Pebesma 2018), accounting for each transect's linear extent. We created a binary spatial‐neighbor matrix using the minimum distance in the spdep package (Bivand 2022) at which all transects had at least one neighbor (210 m) and row‐standardized the weights. Moran's *I* was used with 9999 Monte Carlo permutations to test for positive spatial autocorrelation. Sensitivity analyses were carried out using neighborhood thresholds of 260, 310, 360, and 410 m.

We used the tree use versus availability framework (Manly et al. 2002; Tamang et al. 2024) using the “widesI” function in the adehabitatHS package (Calenge and Basille 2023) to analyze tree selection by woodpeckers in order to determine whether specific tree species were used in a comparable proportion to availability, selected (utilized in a greater amount in comparison to availability), or avoided (utilized in a smaller amount in comparison to availability). The distribution of all continuous variables was checked using the Shapiro–Wilk normality test. Tree DBH and tree height were not normally distributed (Tree DBH: Shapiro–Wilk normality test = 0.958, *p* = 0.009; Tree height: Shapiro–Wilk normality test = 0.961, *p* = 0.014), so the Wilcoxon rank‐sum test (*W*) (Haynes 2013) was employed to analyze differences in tree attributes between the nesting and non‐nesting trees. However, an independent two‐sample *t*‐test (*t*) (Ross and Willson 2017) was used for canopy cover data, which was normally distributed (Shapiro–Wilk normality test = 0.986, *p* = 0.491), and homogeneity of variances was also satisfied based on Levene's test (*F* = 1.147, *p* = 0.288).

We used a multicollinearity test among all the predictor variables influencing cavity‐nest occurrence. The variables tree height (*r* = 0.84) and distance to water (*r* = −0.99) were removed as they had a correlation coefficient greater than |*r*| > 0.7 with tree DBH and distance to settlement, respectively. Retention of tree DBH was done as it is a direct measurement of wood amount and structural capacity, and distance to settlement represents the potential anthropogenic disturbance and use of forest resources. The final analysis only retained tree DBH, canopy cover, distance to forest edge, distance to settlement, and living condition. Before modeling, all continuous variables were standardized using the *z*‐score transformation to improve model interpretability. We employed a Generalized Linear Mixed Model (GLMM) with a binary response variable that was used to identify factors affecting the cavity nesting presence of woodpeckers. Transect was included as a random variable to account for repeated surveys conducted in the same transects. We performed model selection using an information‐theoretic approach and ranked the model combinations using Akaike information criterion adjusted for small sample size (AICc) (Burnham and Anderson 2002). The model selection was done using the “dredge” function in the MuMIn package (Barton 2012), and the models with ΔAICc ≤ 2 were averaged for the GLMM.

## 3 Results

### 3.1 Woodpecker Species Community Structure

A total of 51 individuals from nine woodpecker species were recorded in the Pathlaiya CF. Distance sampling estimated the mean abundance and density of woodpeckers from each independent visit in the Pathlaiya CF to be 126.39 ± 16.94 individuals and 0.54 ± 0.07 birds/ha, respectively, with a detection probability of 0.79 ± 0.05 (Table 1). The Black‐rumped Flameback was the most abundant woodpecker species reported, accounting for 27.5% of all sightings (*n* = 14), followed by Gray‐headed woodpecker, Rufous woodpecker, and Lesser yellownape (Figure 2). Greater Yellownape *Chrysophlegma flavinucha* , on the other hand, was reported only once. There was no significant positive spatial autocorrelation detected at the primary 210 m threshold in the residuals of the distance model using Moran's I for visit one (*p =* 0.349), visit two (*p* = 0.157), and residuals averaged across both visits (*p =* 0.23; Table 2). Moreover, no significant positive spatial autocorrelation was detected at any of the alternative neighborhood thresholds examined for sensitivity analysis (Table S3).

**TABLE 1:** Distance sampling analyses of the overall woodpecker species recorded. The selected top model, with the lowest AIC value, consists of a hazard rate with no cosine adjustment, estimating woodpecker detection probability, covered region abundance, total abundance, and density.

Parameter	Estimate	SE	CV %	95% confidence interval
Detection probability	0.79	0.05	7.51	—
Covered region abundance	64.03	6.37	9.9	—
Total abundance (Visit 1)	112.44	18.76	16.6	[80.57–156.92]
Total abundance (Visit 2)	140.35	27.18	19.3	[95.18–206.95]
Total abundance (total mean)	**126.39**	**16.94**	**13.3**	**[96.9–164.87]**
Density (birds per hectare) (Visit 1)	0.48	0.08	16.6	[0.35–0.68]
Density (birds per hectare) (Visit 2)	0.6	0.11	19.3	[0.41–0.89]
Density (birds per hectare) (Total mean)	**0.54**	**0.07**	**13.3**	**[0.42–0.71]**

*Note:* Bold indicates the mean values from both visits.

Abbreviations: CV, coefficient of variation; SE, standard error.

**FIGURE 2:**
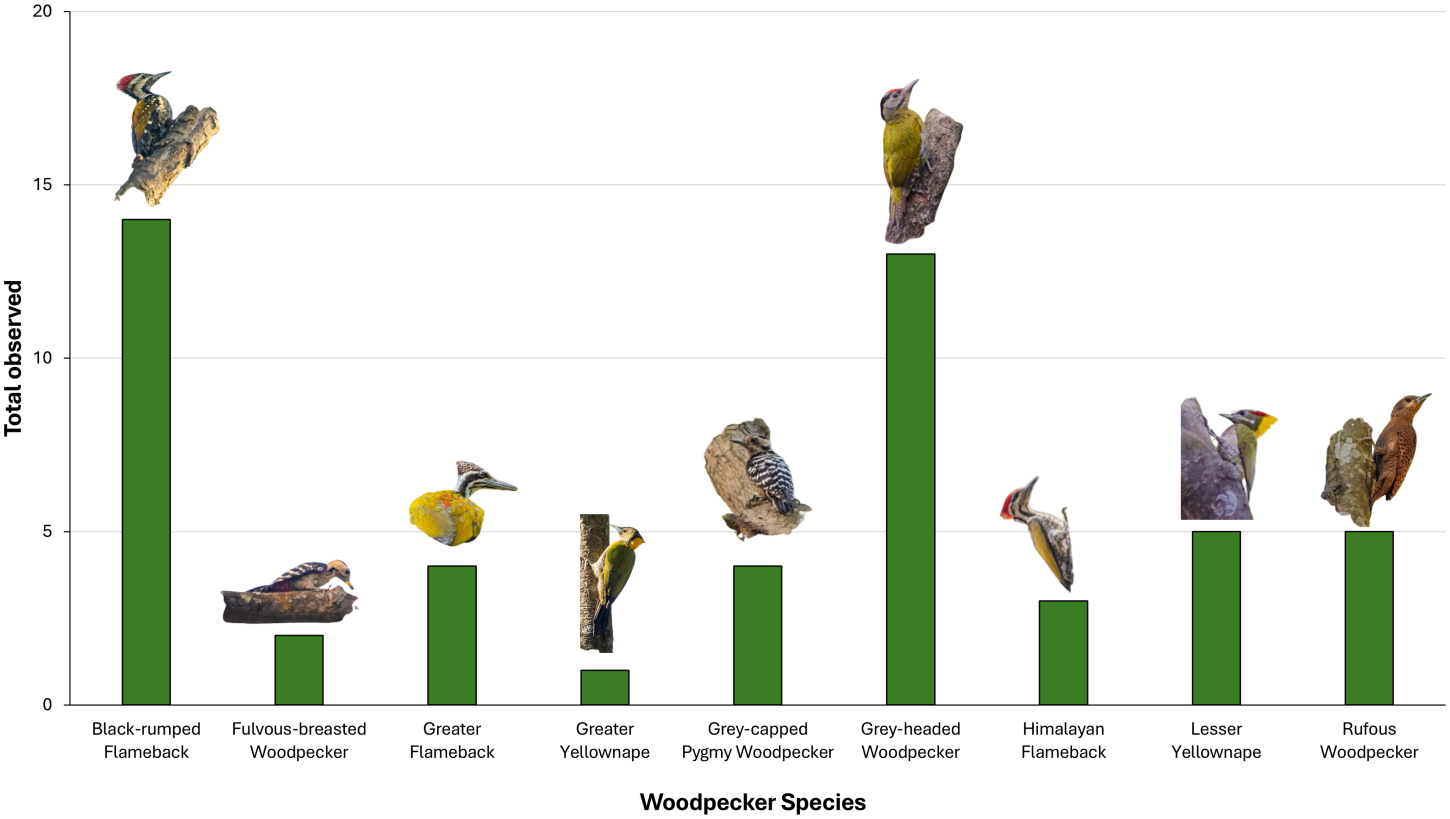
Total woodpecker species observed in Pathlaiya CF with their abundance.

**TABLE 2:** Spatial autocorrelation of the distance model residuals.

Residual dataset	Distance threshold (m) (minimum distance neighbor)	Moran's *I*	*p*
Visit 1	210	0.026	0.349
Visit 2	210	0.147	0.157
Total mean	210	0.099	0.230

### 3.2 Nesting Tree Preference and Characteristics

We recorded a total of 11 different species of trees in Pathlaiya CF, out of which only five species had a woodpecker nesting cavity. Two tree species were used more frequently for nesting cavities compared to their availability: sal, *Shorea robusta*, and jamun, *Syzygium cumini*, with a higher preference for sal (Table 3). Two species, kusum, *Schleichera oleosa*, and karma, *Adina cordifolia*, were used in proportion to their availability. Tatari, *Dillenia pentagyna*, was the only species that was used less often than the nesting‐cavity availability, whereas no nesting cavities were found in bot dhairo, *Lagerstroemia parviflora*; sindure, *Bixa Orellana* ; simal, *Bombax ceiba* ; seto siris, *Albizia procera* ; teak, *Tectona grandis* ; and satisal, *Dalbergia latifolia* . Overall, Manly's selection ratio analysis revealed significant non‐random tree species selection by woodpeckers (*χ* ^2^ = 23.55, df = 10, *p* = 0.009) in the Pathlaiya CF.

**TABLE 3:** Woodpecker nesting cavity tree preference according to the use versus availability framework in Pathlaiya CF.

Tree species	Scientific name	Available (%)	Nest use (%)	Selection ratio (Wi)	SE (Wi)	Preference	*p*	Bi
Sal	***Shorea robusta***	**29.3**	**61.3**	**2.09**	**0.47**	**+**	**0.019**	**0.329**
Jamun	*Syzygium cumini*	7.3	9.7	1.32	0.89	+	0.718	0.208
Kusum	*Schleichera oleosa*	6.1	6.5	1.06	0.86	0	0.946	0.167
Karma	*Adina cordifolia*	19.5	19.4	0.99	0.43	0	0.985	0.156
Tatari	*Dillenia pentagyna*	3.7	3.2	0.88	1	−	0.906	0.139
Bot Dhairo	*Lagerstroemia parviflora*	13.4	0	0	0.01			
Sindure	*Bixa orellana*	9.8	0	0	0.01			
Simal	*Bombax ceiba*	4.9	0	0	0.02			
Seto Siris	*Albizia procera*	2.4	0	0	0.04			
Teak	*Tectona grandis*	2.4	0	0	0.04			
Satisal	*Dalbergia latifolia*	1.2	0	0	0.08			

*Note:* Trees utilized more relative to availability (values of Wi > 1; +), used in proportion to availability (value of Wi = 1; 0) and used relatively less than availability (value Wi < 1; −). Blanks represent tree species not used for nesting. P: Standard Selection Ratio and Bi: Bonferroni‐adjusted standardized selection ratio. Bold indicates significant value.

In total, we sampled 82 trees in the study area, which included 31 nesting trees and 51 non‐nesting trees. All nesting tree characteristics differed significantly from those of non‐nesting trees, and nesting trees had greater canopy cover, larger tree DBH, and greater tree height (Figure 3). Canopy cover varied significantly between nesting and non‐nesting trees (*t* = −6.283, df = 80, *p* < 0.001). Tree DBH (*W* = 359, *p* < 0.001) and tree height (*W* = 276, *p* < 0.001) were also significantly different between nesting and non‐nesting trees. Nesting trees had a greater mean canopy cover of 68.55% ± 7.86% (median = 69.01%; range = 53.99%–86.01%); meanwhile, non‐nesting trees had a mean canopy cover of 58.39% ± 6.60% (median = 58%; range = 40.01%–72%). Similarly, tree DBH (nesting tree = 187.58 ± 55.85 cm, non‐nesting trees = DBH of 131.25 ± 59.35 cm) and tree height (nesting trees = 15.48 ± 2.38 m, non‐nesting trees = 11.14 ± 3.82 m) were also relatively higher in nesting trees. Additionally, the mean height of nesting cavities from ground level was found to be 9.7 m, and most of the nesting cavities (*n* = 8) were oriented toward the south.

**FIGURE 3:**
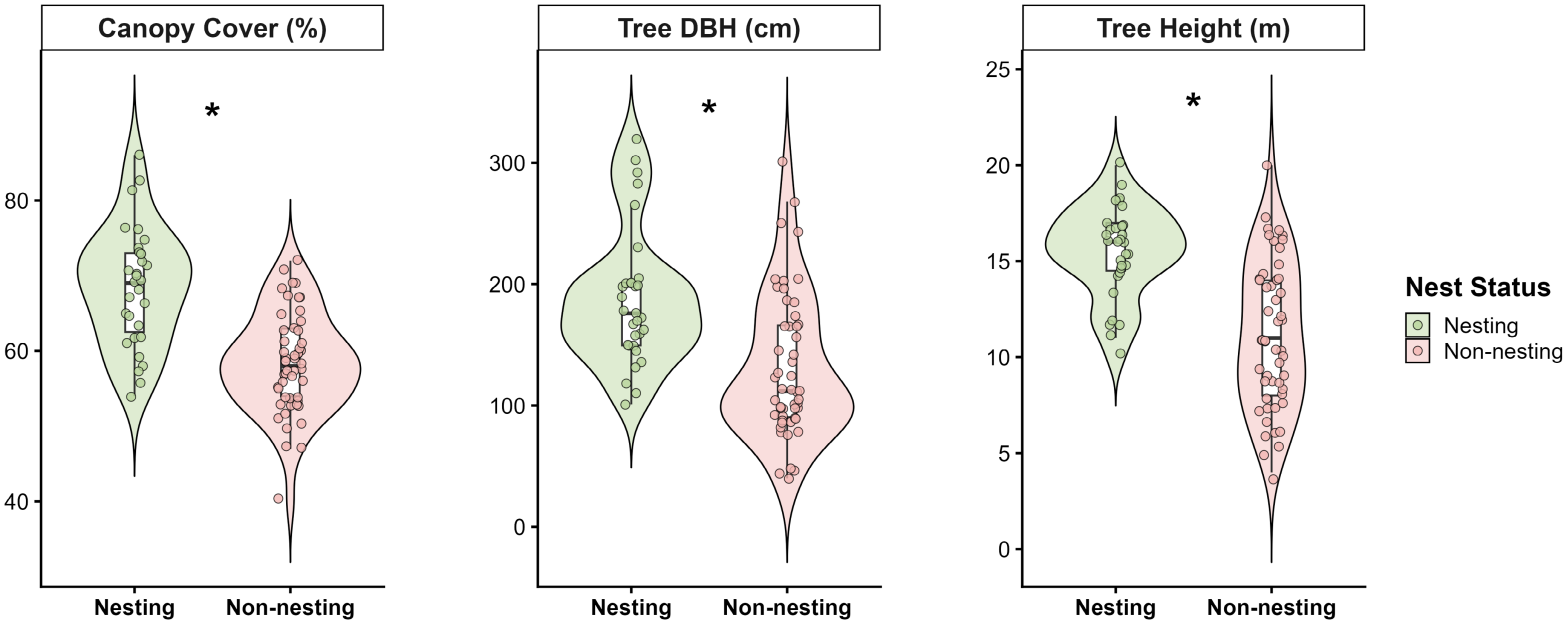
Comparison of nesting and non‐nesting tree characteristics of the woodpecker in the Pathlaiya CF. Astericks (*) indicate statistically significant differences (*p* < 0.05).

### 3.3 Factors Influencing the Presence of a Woodpecker Nesting Cavity

The model selection was performed with factors that impact the occurrence of the woodpecker nesting cavity, and the top‐ranked model with ΔAICC ≤ 2 were averaged coefficients as the best‐fit model explaining the nesting occurrence (Table 4). The best model incorporated canopy cover, tree DBH, and distance to forest edge, which had a positive impact on nesting occurrence, and tree condition and distance to settlement, which had a negative effect (Table 5). The top models averaged coefficients for only three parameters: canopy cover (*p* < 0.001), tree condition (*p* < 0.05), and tree DBH (*p* < 0.05), which significantly influenced woodpecker cavity‐nest occurrence.

**TABLE 4:** Top‐ranked Generalized Linear Mixed Models (GLMM) with ΔAICc ≤ 2 describing the parameters affecting woodpecker cavity‐nest occurrence in Pathlaiya CF.

Model components	df	loglik	AICc	Delta	Weight
Canopy cover + Tree condition + Tree DBH	5	−26.9	64.6	0	0.366
Canopy cover + Tree condition + Tree DBH + Distance to forest edge	6	−25.9	65.04	0.45	0.293
Canopy cover + Tree condition + Tree DBH + Distance to settlement	6	−26.5	66.11	1.51	0.172
Canopy cover + Tree condition + Tree DBH + Distance to forest edge + Distance to settlement	7	−25.4	66.35	1.75	0.152

**TABLE 5:** GLMM with a binary response variable of the full model‐averaged coefficients (ΔAICc ≤ 2) influencing the woodpecker cavity‐nest occurrence in Pathlaiya CF.

Parameters	Estimate	SE	Adjusted SE	*z* value	*p*
Intercept	0.314	0.476	0.484	0.649	0.516
Canopy cover (%)	1.642	0.486	0.493	3.327	**< 0.001**
Tree condition (Living)	−2.466	0.789	0.801	−3.078	**0.002**
Tree DBH (cm)	1.309	0.435	0.367	2.964	**0.003**
Distance to forest edge (m)	0.235	0.364	0.367	0.642	0.521
Distance to Settlement (m)	−0.131	0.306	0.309	−0.425	0.671

*Note:* Bold indicates significant values.

## 4 Discussion

Our study found nine species of woodpeckers in the Pathlaiya CF, which account for 36% of the total species of woodpeckers in Nepal, depicting the importance of the sub‐tropical sal‐dominated landscape for primary cavity excavators, especially woodpeckers. Although the species richness of our study was lower compared to the studies from the tropical rainforests of Southeast Asia (Lammertink 2004; Styring and Ickes 2001), which is a global hotspot for tree cavity nesting birds (van der Hoek et al. 2017), the species richness was comparatively higher than the study conducted in the sub‐Himalayan moist temperate woods of India (Menon and Shahabuddin 2021). This highlights the importance of Nepal's Terai sal‐dominated forest as a dynamic habitat for forest‐dependent and cavity‐nesting birds, given that woodpeckers are indicators of overall forest bird richness (Drever et al. 2008).

Our study revealed a woodpecker density of 0.42–0.71 birds/ha, which is consistent with a study conducted using distance sampling in the South Asian region in the Sub‐Himalayan sal forests of North‐west India (Kumar et al. 2014), where woodpecker density range from 13.8 to 74.5 km^−2^ (equivalent to 0.138 to 0.745 birds/ha), suggesting broad comparable woodpecker densities within sal‐dominated forests of the region. Studies in the rainforests of Southeast Asia (Styring and Ickes 2001), North America (Drever et al. 2008), and temperate forests of Europe (Domokos and Cristea 2014) found woodpecker density to be 0.78 ± 0.29 birds/ha in unlogged forests, 0 to 0.9 birds/ha, and 0.053 birds/ha (0.53 per 10 ha), respectively, which varies compared to our study. This variation shows the role of different forest types, management, history, resource availability, and composition globally in supporting woodpecker species richness. The highest observation of the Black‐rumped Flameback may be related to its biological character as a habitat generalist (Menon and Shahabuddin 2021), since it is often seen in human‐modified habitats, orchards, and forest edges (Kumar et al. 2014; Menon and Shahabuddin 2021). In contrast, Greater Yellownape, a sensitive bio‐indicator of mature, complex forests, has a high probability of occurrence when canopy cover exceeds 50% (Menon and Shahabuddin 2021), which could explain the low observation rate, as the Pathlaiya CF has human‐modified edges that may reduce their occurrence.

The cavity nesting tree preference of woodpecker species according to the tree use versus availability framework shows woodpeckers preferred two tree species out of 11 tree species (Table 3) that were sampled in the study area. The preference for two species, sal and jamun, is consistent with a previous study in Nepal's lowland forests (Baral et al. 2018), where these species have been identified as important cavity‐bearing tree species, indicating woodpeckers' preference for cavity nesting. The tree species preferred by woodpeckers in Pathlaiya CF were mostly hardwoods, which are difficult to excavate but provide excellent safety and durability as nesting resources (Jackson and Jackson 2004). Despite their physical hardness, some species may be preferred because they provide predator resistance and superior thermal insulation for nestlings (Hebda et al. 2017). Furthermore, even hardwoods possessing high physical toughness, such as sal, are susceptible to heart rot fungi, which are easily detected by woodpeckers, allowing them to avoid the hard exterior sapwood and excavate a hollow in the softer interior (Jackson and Jackson 2004). The avoidance of simal and sindure for nesting cavities in our study might be due to their softwood status, which can cause rapid decay or lack the structural partitioning needed to create a stable, long‐lasting cavity. However, teak is a hardwood species, and the avoidance of woodpeckers for nesting cavities in this species is consistent with observations from Borneo (Sheldon et al. 2010) and India (Kumar et al. 2014), where exotic plantations (Teak is also an exotic species in Nepal) support lower woodpecker richness, most probably due to a lack of rough‐barked foraging substrates and a lower susceptibility to the specialized decay processes required for excavation. In the lowlands of Nepal, increasing exotic species plantations of high‐value timber species, such as teak, are replacing the native sal forest, as they provide high economic benefit; however, this is detrimental to the overall ecosystem and biodiversity.

The cavity nest entrance orientation shows that they were distributed among several directions, limiting evidence for strong directional preference. Woodpeckers preferred mature forest stands with larger DBH and greater canopy cover which were positively associated with cavity nest occurrence. Trees with a greater diameter provide enough wood volume to create cavity nests and provide a variety of microhabitat features, such as cracks and bark (Vuidot et al. 2011), which increases the availability of wood‐boring insects that woodpeckers consume. In tropical sal forests of Nepal, tree DBH is a significant predictor of both natural and excavated cavity existence (Bhusal et al. 2016), which coincides with our data. Additionally, the mean nesting cavity height in our study was 9.7 m, which is similar to the findings from a study in lowland Nepal, where they found a positive correlation between nesting cavity height and tree DBH (Baral et al. 2018). These benefits provided by larger trees might clarify their cavity‐nesting preference. Nesting preference in greater canopy cover might be explained by the advantages, such as maintaining stable microclimatic conditions, which are necessary for nestling development (Sekercioglu et al. 2002). Woodpeckers were found to prefer larger snags (standing 3D: Dead, Decayed and Damaged) over healthy live trees, as snags are easier to excavate, require less effort (Jackson and Jackson 2004), and expedite foraging in woodpeckers (Drever et al. 2008). The density of snags in the sub‐Himalayan sal forests has been revealed to strongly impact overall woodpecker population and species richness, especially important during the breeding season, when energy‐rich food requirements are highest (Kumar et al. 2014). This not only provides them with food but also exposes the underlying substrate to other insectivorous bird species. Nesting success is also increased when woodpeckers use older and reused nests, which are frequently found in snag‐dense environments (Pakkala et al. 2017). These findings have significant implications for retention‐based forest management, notably the preservation of mature native trees, snags, and cavity‐bearing tree species in South Asian forest landscapes.

Woodpeckers' preferences for snags and larger trees with dense canopy coverage area for cavity nesting (Figure 4) show their reliance on forests with such tree characteristics and conditions; however, in managed forests, such trees are removed first, making the woodpecker population extremely vulnerable. Pathlaiya CF's operation plan allows for an Irregular Shelterwood silvicultural management strategy, which involves removing a set number of old, bigger trees to produce an uneven stand structure (Raymond and Bédard 2017) and the removal of selected dead, dying, diseased, or depressed wood. This is a balanced approach for forest management in the tropical sal forests of Nepal; however, there are various silvicultural practices, such as clear‐felling, which have been applied in the lowlands of Nepal. These practices potentially leads to an increased threat to cavity nesters, especially woodpeckers, when the tree species with appropriate characteristics for foraging and nesting are removed. It has been found that woodpecker abundance may follow a ‘law of diminishing returns’, which states that exponentially increasing the number of trees used by woodpeckers does not lead to increased abundance; however, if the abundance of preferred trees (snags) falls below a certain threshold, woodpecker abundance will decline (Gunn and Hagan Iii 2000). However, it is not always true in terms of forest management issues, since it also increases fire susceptibility, as woodpeckers mostly prefer snags. Moreover, below a certain threshold, there may be a favorable relationship between snag availability and woodpecker population, but having an excessive quantity of snags for species conservation might have a detrimental influence on overall forest species (Gunn and Hagan Iii 2000). Therefore, we would like to stress that, for proper forest management and not only cavity nester conservation but overall forest biodiversity, it is necessary to identify the species' preferred nesting trees and implement management strategies that account for the possible release of such live trees and snags for cavity substrate from harvest in appropriate amounts.

**FIGURE 4:**
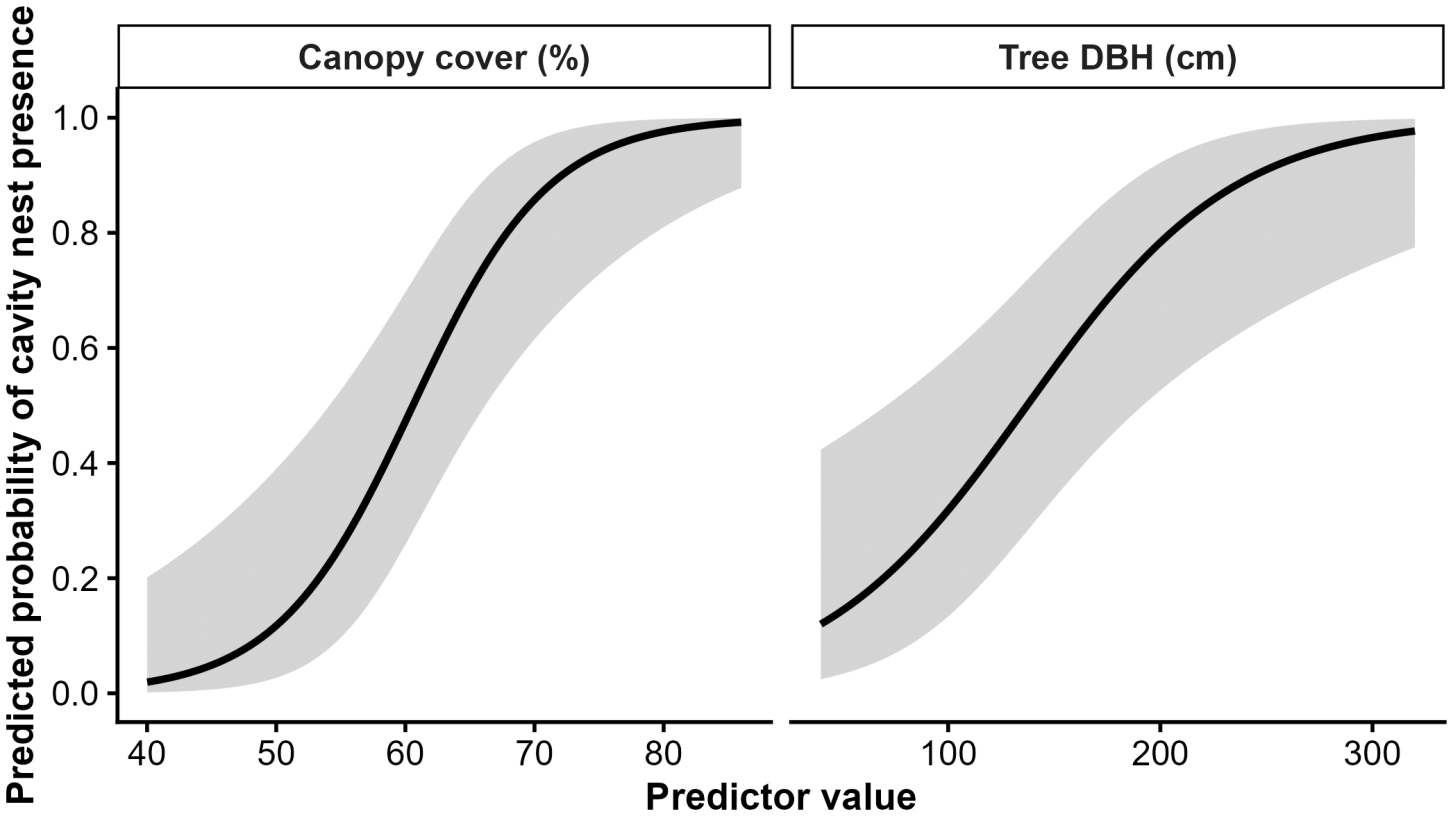
Predicted probability of continuous variables canopy cover and tree DBH influence on the cavity nest presence of woodpeckers in Pathlaiya CF.

## 5 Conclusion

Woodpeckers are keystone species that play a vital role in forest ecosystems, and studies focusing on this taxon can help understand overall forest bird diversity and forest health. Our study fills a critical void in woodpecker species richness and nesting ecology in sub‐tropical forests of South Asia, where such information is crucial for biodiversity conservation and forest management. The sal forest of lowland Nepal supports a multitude of woodpecker species with their preferred nesting tree species available, highlighting the conservation value of these landscapes for primary cavity excavators in South Asia. Woodpeckers' non‐random preference for tree characteristics in cavity nesting shows that they are highly dependent on mature forests, with tall trees, larger DBH and snags. This specific preference of the species puts them at high vulnerability, as trees with such characteristics are removed foremost in forest management practices. Therefore, the forest management plan should incorporate partial retention of such preferred species by the primary cavity excavators, as they are of high conservation concern, playing a vital role in the forest ecosystem, supporting various other organisms. Nepal is a global leader in community forest management practices, which incorporate the participation of local stakeholders in forest management and conservation activities. Our study in the Pathlaiya CF emphasizes the role of the community forests in biodiversity conservation and provides inputs to improve management practices, which can help the cavity nester population flourish in the long term. Similar studies in the temperate Himalayan regions of South Asia can further enhance our knowledge of the species' ecology and the importance of the diverse Himalayan landscape for primary cavity excavators.

## Author Contributions

**Nikeet Pradhan:** conceptualization (equal), data curation (equal), formal analysis (equal), investigation (lead), methodology (equal), visualization (lead), writing – original draft (equal), writing – review and editing (equal). **Sandeep Regmi:** data curation (equal), formal analysis (equal), writing – review and editing (equal). **Bikash Adhikari:** conceptualization (equal), methodology (equal), writing – review and editing (equal).

## Funding

The authors have nothing to report. Field equipment was supported by Idea Wild, USA.

## Ethics Statement

The research permission in the Pathlaiya CF was provided by the Division Forest Office, Bara District, Nepal, and the Pathlaiya Community Forest Users' Group.

## Conflicts of Interest

The authors declare no conflicts of interest.

## Supporting information


**Figure S1:** Detection perpendicular distance of woodpeckers from the line transects.
**Figure S2:** Distance sampling goodness of fit: Cramer‐von Mises test (unweighted) (Test statistic = 0.0075, *p* = 0.72).
**Table S1:** Distance sampling analysis modeling with three key functions to find the lowest AIC model for estimation of abundance, density and detection probability.
**Table S2:** Summary statistics of survey effort and encounter rates for each independent visit for distance sampling analysis.
**Table S3:** Sensitivity of the distance model residuals to alternative neighborhood thresholds using Moran's *I*.

## Data Availability

All data from the study have been made available via the Zenodo online data service. URL: https://doi.org/10.5281/zenodo.20830364.
