# Supplementary material for: Primary Cavity Excavators in Community‐Managed Lowland Forest of Nepal: Woodpecker Assemblage, Nest‐Tree Preference, and Its Implications for Forest Management
Source: Ecol Evol. 2026 Nov 10;16(11):e74611. doi: 10.1002/ece3.74611 (PMC13750262; doi:10.1002/ece3.74611)
Supplement: Supplementary file 1 [file ECE3-16-e74611-s001.docx]

**Supplementary Information**

Figure S1: Detection perpendicular distance of woodpeckers from the line transects.

Table S1: Distance sampling analysis modeling with three key functions to find the lowest AIC model for estimation of abundance, density and detection probability.

| S.N. | Model | Key Function | C-vM p-value | Detection probability | SE | df | AIC | AICc |
| --- | --- | --- | --- | --- | --- | --- | --- | --- |
| 1 | Hazard-rate | Cosine adjustment (order 1) | 0.72 | 0.796 | 0.06 | 2 | 391.3 | 0 |
| 2 | Hazard-rate | Simple polynomial (orders 2,4) | 0.72 | 0.796 | 0.06 | 2 | 391.3 | 0 |
| 3 | Hazard-rate | Hermite polynomial (orders 2,4) | 0.72 | 0.796 | 0.06 | 2 | 391.3 | 0 |
| **4** | **Hazard-rate** | **Null** | **0.72** | **0.796** | **0.06** | **2** | **391.3** | **0** |
| 5 | Uniform | Simple polynomial (orders 2,4) | 0.610 | 0.736 | 0.063 | 3 | 393.55 | 2.221 |
| 6 | Uniform | Hermite polynomial (orders 2,4) | 0.610 | 0.736 | 0.063 | 3 | 393.55 | 2.221 |
| 7 | Half-normal | Cosine adjustment (order 1) | 0.522 | 0.711 | 0.113 | 1 | 393.2 | 2.23 |
| 8 | Half-normal | Simple polynomial (orders 2,4) | 0.522 | 0.711 | 0.113 | 1 | 393.2 | 2.23 |
| 9 | Half-normal | Hermite polynomial (orders 2,4) | 0.522 | 0.711 | 0.113 | 1 | 393.2 | 2.23 |
| 10 | Half-normal | Null | 0.522 | 0.711 | 0.113 | 1 | 393.2 | 2.23 |
| 11 | Uniform | Cosine adjustment (order 1) | 0.549 | 0.713 | 0.107 | 3 | 395.5 | 4.2 |

Table S2: Summary statistics of survey effort and encounter rates for each independent visit for distance sampling analysis

n: total observations

k: total transects

er: Encounter Rate

| Region | Area | Covered Area | Effort | n | k | ER | se.ER | cv.ER |
| --- | --- | --- | --- | --- | --- | --- | --- | --- |
| 1 | 232.14 | 58.8 | 6 | 26 | 24 | 4.3 | 0.6 | 0.1 |
| 2 | 232.14 | 58.8 | 6 | 25 | 24 | 4.2 | 0.7 | 0.2 |
| Total | 464.28 | 117.6 | 12 | 51 | 48 | 4.3 | 0.5 | 0.1 |

Figure S2: Distance sampling goodness of fit: Cramer-von Mises test (unweighted) ( Test statistic = 0.0075, p-value = 0.72).

Table S3: Sensitivity of the distance model residuals to alternative neighborhood thresholds using Moran’s I.

| Residual dataset | Distance threshold (m) (minimum distance neighbor) | Moran’s I | P value |
| --- | --- | --- | --- |
| Visit 1 | 260 | 0.007 | 0.363 |
| Visit 2 | 260 | 0.072 | 0.226 |
| Total mean | 260 | 0.004 | 0.373 |
| Visit 1 | 310 | -0.04 | 0.473 |
| Visit 2 | 310 | 0.034 | 0.282 |
| Total mean | 310 | -0.048 | 0.373 |
| Visit 1 | 360 | -0.138 | 0.753 |
| Visit 2 | 360 | -0.042 | 0.476 |
| Total mean | 360 | -0.075 | 0.578 |
| Visit 1 | 410 | -0.036 | 0.443 |
| Visit 2 | 410 | -0.042 | 0.476 |
| Total mean | 410 | -0.031 | 0.431 |
